# Orthostatic Hypertension: Clinical Spectrum and Gaps in Knowledge

**DOI:** 10.1007/s11906-026-01386-3

**Published:** 2026-09-16

**Authors:** David Moloney, Jens Jordan, Italo Biaggioni

**Affiliations:** 1 Vanderbilt Autonomic Dysfunction Center, Division of Genetic Medicine and Clinical Pharmacology, Department of Medicine, Vanderbilt University Medical Center, 560 RRB, VUMC, 2222 Pierce Ave, Nashville, TN USA; 2 Institute of Aerospace Medicine, German Aerospace Center (DLR), Cologne, Germany; 3 Medical Faculty, University of Cologne, Cologne, Germany

**Keywords:** Orthostatic hypertension, Autonomic nervous system, Sympathetic nervous system, Posture, Hypertension

## Abstract

**Purpose of Review:**

Orthostatic hypertension (oHT) is increasingly recognized as a clinically relevant but poorly understood disorder of autonomic blood pressure control. This review aims to summarize the current understanding of the clinical spectrum and pathophysiology of oHT, evaluate its prognostic significance, and identify key gaps in knowledge to guide future mechanistic and clinical studies.

**Recent Findings:**

Recent evidence has shifted the perception of oHT from a physiological variant to a condition associated with adverse outcomes. In younger adults, oHT has been linked to incident hypertension, while in older populations it is associated with cardiovascular, cerebrovascular, renal, and cognitive complications. Excessive sympathetically mediated vasoconstriction appears to be the predominant mechanism, although arterial stiffness, impaired baroreflex buffering, and abnormal vascular responsiveness may also contribute depending on the population. Consensus definitions have recently standardized diagnostic criteria, but uncertainties remain regarding optimal thresholds, measurement reproducibility, and the mechanisms underlying its heterogeneous presentation.

**Summary:**

Despite growing recognition, important questions remain regarding whether oHT represents an independent cardiovascular risk factor or primarily a marker of vascular aging and hypertension. Evidence supporting oHT-specific therapeutic strategies is currently lacking. Further research is needed to clarify its pathophysiology, refine diagnostic approaches, and determine its clinical and prognostic significance.

## Introduction

Upright posture imposes significant gravitational stress on the cardiovascular system requiring rapid and coordinated autonomic nervous system adjustments to maintain arterial blood pressure. Upon standing, gravity induces venous pooling in the splanchnic circulation and lower extremities, resulting in an abrupt reduction in venous return, decreased stroke volume, and a transient blood pressure fall. These hemodynamic changes unload stretch sensitive carotid and aortic baroreceptors, leading to reduced afferent signaling to brainstem cardiovascular regulatory nuclei. Central command and input from other sensory systems like cardiopulmonary receptors and the vestibular organs modulate the autonomic response. Heart rate increases within 1–2 s due to parasympathetic withdrawal while sympathetic activation increases peripheral vasoconstriction over approximately 5–10 s [1]. After the first 15–30 s, upright blood pressure is maintained primarily by sympathetically-mediated vasoconstriction and to a lesser degree by increased heart rate and cardiac contractility. Renal sympathetic activation, through direct tubular actions that increase renal sodium reabsorption and activation of the renin-angiotensin-aldosterone system, contribute to maintenance of longer-term upright blood pressure, but do not play an important role in the immediate (1–10 min) response to upright posture, when oHT is often defined.

The importance of these compensatory autonomic responses for blood pressure control is particularly evident when they fail, leading to neurogenic orthostatic hypotension. The clinical significance of orthostatic hypotension is obvious in patients that have disabling symptoms of cerebral hypoperfusion, and increased risk of syncope and falls. In addition, large epidemiological studies have shown that orthostatic hypotension is associated with increased risk of cardiovascular disease, heart failure, stroke and cognitive impairment, and, importantly, is an independent risk factor for mortality [2]. 

**Fig. 1:**
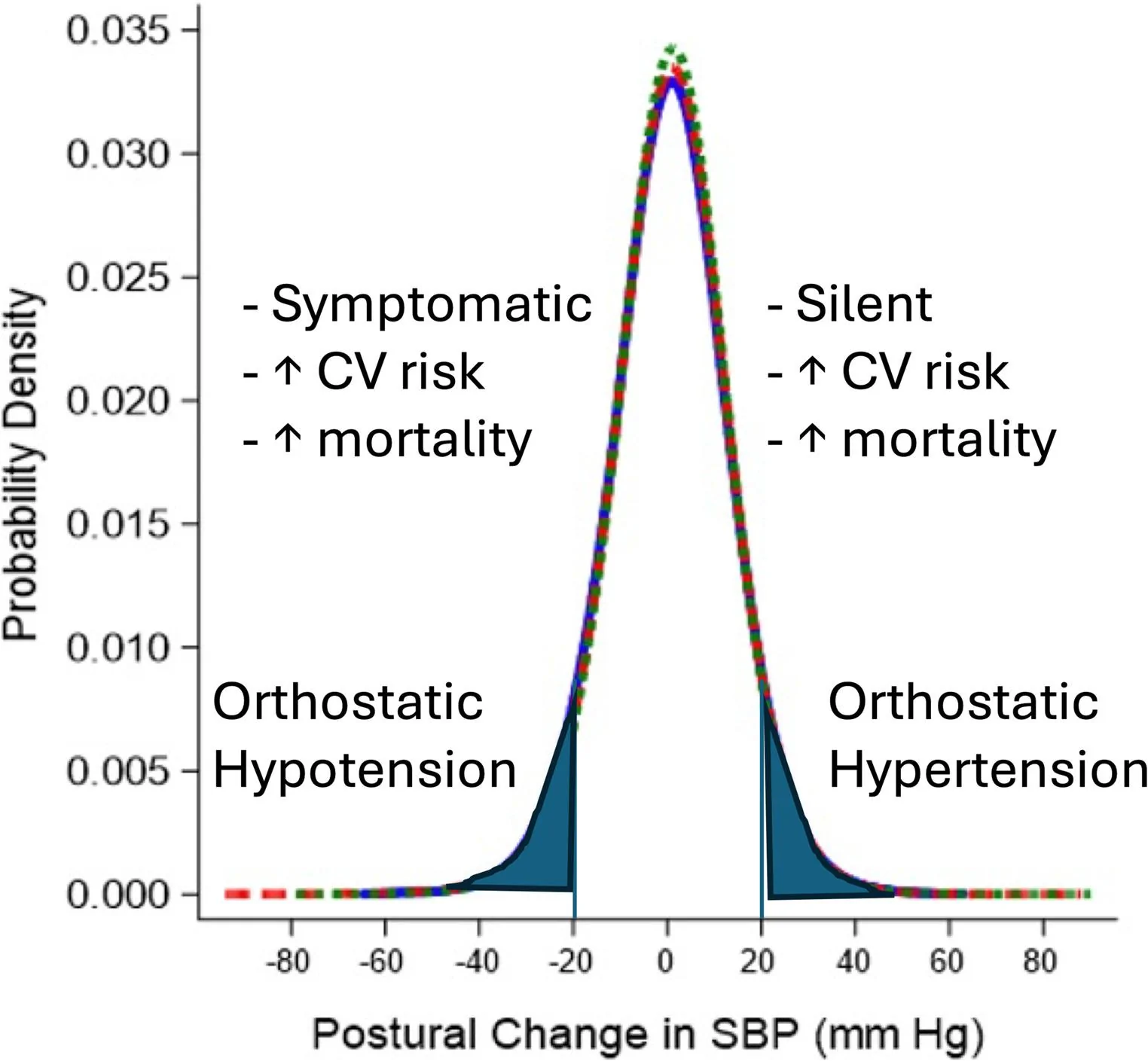
Frequency distribution (y-axis) of the postural change in systolic blood pressure (SBP, x-axis), with orthostatic hypotension and orthostatic hypertension on both ends of the normal distribution. Both extremes have been associated with increased risk of adverse cardiovascular outcomes and mortality. Figure adapted from [32]

At the opposite end of the orthostatic blood pressure spectrum are individuals with a marked, sustained pressor response to standing (Fig. 1). Once this pressor response exceeds a diagnostic threshold (detailed below), the phenomenon is referred to as orthostatic hypertension (oHT). oHT was initially identified in small physiological studies aimed at characterizing individual variability in cardiovascular adaptation to upright posture [3–5]. Because it is largely asymptomatic in most individuals, oHT received considerably less attention than orthostatic hypotension. Paradoxically, its clinical relevance was recognized only after orthostatic cardiovascular data became available from epidemiological studies originally designed to study orthostatic hypotension. When investigators analyzed the clinical implications of oHT, they found it to be linked to a higher risk of cardiovascular disease, cerebrovascular disease, kidney disease, cognitive impairment, and all-cause mortality [6–8]. 

In this review, we summarize the current understanding of the clinical spectrum and pathophysiology of oHT, examine prognostic implications, and highlight knowledge gaps that could guide future mechanistic and clinical studies.

## Diagnostic Criteria

The recent consensus statement has provided two ways to conceptualize an abnormal orthostatic blood pressure rise: an exaggerated orthostatic pressor response, defined by a sustained rise in systolic blood pressure of at least 20 mm Hg on standing, and oHT, defined as an exaggerated orthostatic pressor response associated with an absolute systolic blood pressure of at least 140 mmHg while standing [9]. 

The exclusion of diastolic blood pressure in these consensus criteria was based on physiologic, epidemiologic, and practical considerations. Upright diastolic blood pressure is strongly affected by sympathetically-mediated vasoconstriction, and by the increase in heart rate, which reduces the time available for diastolic runoff. Thus, diastolic blood pressure normally increases with upright posture, making it difficult to establish an abnormal threshold. Also, most of the epidemiological evidence linking oHT with adverse outcomes is based on systolic blood pressure values.

It should be noted that, in addition to these definitions, there is a body of literature focusing on observations in young adults that smaller orthostatic blood pressure increases predict future incident hypertension and cardiovascular outcomes [10–12]. 

## Prevalence and Associated Clinical Conditions

The prevalence of oHT depends heavily on the definition applied [13], age, presence of hypertension, and measurement protocol. For example, blood pressure measurements from supine to standing will yield other results than those from seated to standing. For these reasons, comparisons between studies are challenging and precise prevalence figures are difficult to ascertain. Nonetheless, both the prevalence and clinical relevance of orthostatic hypertension is closely associated with the presence of essential hypertension and the age of the individual. In general, oHT is uncommon in healthy younger adults, affecting approximately 1–3% of normotensive individuals, but its prevalence increases with age and the presence of hypertension. In community-dwelling older adults, prevalence ranges from approximately 5% to 15% [14], whereas 10–30% of patients with established hypertension exhibit an exaggerated orthostatic pressor response, depending on the population studied and the diagnostic criteria used [15]. Even within hypertensive populations, the prevalence varies with age. oHT is rare in younger hypertensives, found in 0.6–1.2% of the HARVEST cohort [16], and is much more common in older hypertensives, reported in approximately 28% in the PARTAGE cohort [17]. 

Even though most studies have focused on the relation between oHT and hypertension, oHT has also been reported in younger patients with hyperadrenergic syndromes, such as hyperadrenergic postural orthostatic tachycardic syndrome [18, 19], but its prevalence and clinical implications have not been defined.

## Pathophysiology of Orthostatic Hypertension

The pathophysiology of oHT is not fully understood and the current literature is limited to smaller cross-sectional and mechanistic studies. Of those, many were conducted in patient cohorts not meeting current consensus diagnostic criteria. Nevertheless, a substantial body of evidence supports the concept that oHT results from an exaggerated neurovascular response to orthostatic stress, with excessive sympathetic vasoconstriction being the dominant mechanism. Increased vascular stiffness has been suggested to contribute to oHT in older populations, but Kario et al. found that, in 10 patients with oHT aged 60 years or above, the orthostatic BP increase was selectively abolished by alpha-adrenergic blockade with doxazosin, suggesting that vascular alpha-adrenergic activation is a crucial pathophysiologic mechanism of oHT, even in this elderly population [20]. 

Conceptually, oHT may result either from an intrinsically exaggerated sympathetic response to a normal orthostatic hemodynamic stimulus, or from and appropriate compensatory sympathetic response to an excessive hemodynamic burden. In favor of the latter, Streeten et al. suggested that abnormal venous pooling and the resultant central hypovolemia could elicit an exaggerated orthostatic blood pressure response, and further showed that excessive orthostatic norepinephrine release resolved with lower body compression [3]. Remarkably, in this specific patient group, volume expansion improved upright blood pressure while diuretics tended to make matters worse. This study, however, was limited to 12 patients in whom “oHT” was defined by an upright diastolic blood pressure greater than 90 mmHg upright. In contrast, Lee at al found that individuals with oHT had a significant increase in total peripheral resistance on standing despite similar changes in cardiac output and stroke volume compared to controls, consistent with the concept of the exaggerated sympathetic response was the primary mechanism in the setting of normal hemodynamic changes to orthostatic venous pooling [21]. Overall, these observations suggest that the mechanisms driving sympathetic activation in individuals with oHT, which ultimately determine clinical management, show large inter-individual variability. Future pathophysiological studies, using consensus criteria and identifying potential patient subgroups, may help in our understanding of this phenomenon, and influence prospective therapies.

Similarly, excessive sympathetic vasoconstriction could result from increased upright sympathetic activity driving vasoconstriction, or to an exaggerated vascular response to normal sympathetic stimuli. In favor of the former, there are limited studies documenting exaggerated increases in upright sympathetic activity. Hirai et al., recently observed that 7 adults with oHT have significantly elevated muscle sympathetic nerve activity (MSNA) burst frequency (38.4 ± 12.2 vs. 28.3 ± 5.7 bursts/min, *P* < 0.05) and burst incidence (61.1 ± 21.3 vs. 44.6 ± 11.1 bursts/100 heartbeats, *P* < 0.05) at rest when compared to older adults with essential hypertension but without OHT [22]. MSNA, however, was measured only while supine, not while upright. Kario et al. found higher plasma norepinephrine in the upright posture in oHT compared to patients with hypertension but not oHT [20]. Also, higher 24-hour urinary epinephrine has been associated with greater orthostatic SBP responses [16]. The idea that oHT could be a form of blood pressure hyper-responsiveness is supported indirectly by associations with a lower resting office SBP but higher ambulatory BP [10]. This extreme blood pressure variability is also seen in the high prevalence of ‘extreme dipping’ pattern in 24 h ambulatory BP monitoring in oHT [20]. 

Increased vascular stiffness could amplify vascular responses to sympathetic activation. Indeed, vascular stiffness, known to increase with age, is associated with orthostatic blood pressure increases in older cohorts [23]. There are, however, limited direct data about vascular stiffness in patient cohorts strictly characterized by current consensus criteria. In six studies assessing the relationship between oHT and pulse wave velocity, which is closely related to aortic stiffness, only one showed a significant association between pulse wave velocity and oHT [23–28]. Nolde et al. used a 10 mmHg cut-off for systolic oHT and observed a 1.4 m/s difference in pulse wave velocity compared to those with stable orthostatic blood pressure. In contrast, measurements that reflect increased vascular tone, such as augmentation index and systemic vascular resistance index, are raised in oHT, further supporting the idea of an exaggerated orthostatic vasoconstrictive response. Overall, vascular stiffness could contribute to oHT at multiple levels. Stiff arteries could amplify the pressor response to sympathetic vasoconstriction while arterial stiffening in the carotid sinus and aortic arch could impair afferent baroreflex transduction thereby augmenting orthostatic pressor responses. An extreme example of this phenomenon are the rare patients with afferent baroreflex failure [29], in whom orthostatic hypertension can occur. Surprisingly, we are not aware of studies directly measuring baroreflex function in adult populations with oHT.

In summary, current evidence suggest that sympathetically-mediated vasoconstriction is the primary driver of oHT. Several additional factors may play a role, including arterial stiffness, impaired baroreflex buffering, neurohumoral activation, and altered vascular responsiveness, and their relative contribution may vary depending on age and underlying conditions.

## Clinical Relevance of Orthostatic Hypertension

Some studies have reported an association between oHT and symptoms of orthostatic intolerance that are typically only associated with orthostatic hypotension [21, 30], which can impact the quality of life of those patients. In most patients, however, oHT is asymptomatic but clinically important because of its association with an increased risk of cardiovascular events. The magnitude of this risk varies according to age and comorbidities; in younger normotensive adults, oHT appears to predict future incident hypertension; in older adults, oHT is associated with hypertensive target-organ damage, including chronic kidney disease and silent cerebral infarction, as well as an increased risk of cardiovascular events and all-cause mortality.

Regarding the prognostic value of oHT in younger adults, the CARDIA study used a 5 mmHg increase in upright systolic blood pressure to describe an exaggerated orthostatic pressor response. They found that the presence of this exaggerated response, defined by the lower threshold criteria increased the odds of developing future hypertension at 8 years by a ratio of 2.54 [12]. In the HARVEST study, young adults with untreated stage 1 hypertension, the presence of a systolic blood pressure orthostatic rise of 6.5 mmHg was associated with an odds ratio of 2.45 of being diagnosed with masked hypertension at 3 months [11]. A follow-up study of the HARVEST cohort found that this exaggerated orthostatic systolic blood pressure rise of 6.5 mmHg was associated with an increased rate of cardiovascular and renal adverse events at 17.8 years, even when controlling for incident hypertension (hazard ratio, 1.90 [95% CI, 1.06–3.42]) [10]. 

Regarding the prognostic value of oHT in older adults, a systematic review and meta-analysis of 13 papers assessed the association between oHT and mortality, cardiovascular disease, cerebrovascular disease, and heart failure [6]. For oHT, defined as an orthostatic increase of > 20mmHg, the pooled adjusted association with all-cause mortality was modest but statistically significant: HR 1.21 (95% CI 1.05–1.40). For CVD mortality, the pooled estimate was HR 1.39 (95% CI 1.05–1.84), but this came from only two studies and was dominated by one study, so certainty is limited. For stroke/cerebrovascular disease, the pooled unadjusted OR was 1.94 (95% CI 1.52–2.48), suggesting nearly doubled odds. They found no association between systolic oHT and heart failure, but this analysis was likely underpowered. The ARIC study (Atherosclerosis Risk in Communities) has recently published follow-up data of up to 30 years of this cohort showing that orthostatic increases in SBP were not significantly associated with incident coronary heart disease, heart failure, stroke, fatal coronary heart disease and all-cause mortality, but standing SBP ≥ 140 mm Hg was significantly associated with all outcomes [31]. 

Regarding the previous use of DBP cut-offs to define oHT, this is no longer considered pathological. In the MALMO offspring study, an orthostatic DBP rise of 10 mmHg was a normal healthy phenomenon [23]. Similarly, in their systematic review and meta-analysis of oHT and clinical outcomes, Pasdar et al. observed no association between diastolic oHT and adverse clinical outcomes [6]. 

## Relevance to Management

Despite growing evidence that oHT identifies a higher‑risk blood pressure phenotype, clinical data are lacking on whether modifying oHT itself - as opposed to treating hypertension in general - improves outcomes. In their systematic review and individual participant data meta-analysis of 31,124 participants from 9 randomized trials, Juraschek et al. observed that patient randomization to intensive antihypertensive treatment modestly lowered the occurrence of orthostatic hypertension, defined more broadly as an increase in systolic blood pressure of 20 mmHg and/or diastolic blood pressure of 10 mmHg from seating to standing (OR 0.93, 95% CI 0.90 to 0.96) [32]. 

Likewise, it is not clear at present whether there is merit in targeting the sympathetic nervous system in the treatment of oHT, e.g., with central sympatholytics or alpha adrenergic blockers. As mentioned above, in a small mechanistic study, Kario et al. reported that the orthostatic blood pressure increase was abolished by alpha-adrenergic blockade with doxazosin [20]. Hoshide et al. reported that patients with oHT (defined as the highest decile of orthostatic systolic blood pressure response) randomized to add-on doxazosin given at bedtime (in an open label study) had a marked reduction in oHT, and this was associated with a decrease in urinary albumin/creatinine ratio [33]. Finally, in an individual participant data meta-analysis of 506 patients from 3 randomized trials of ultrasound renal denervation, Kirtane et al. reported that individuals who had hypertension and oHT had a greater reduction in their blood pressure post intervention [34]. These studies suggest that targeting sympathetic activation could be an effective approach in the treatment of oHT. However, oHT specific evidence from randomized controlled clinical trials is lacking.

## Gaps in Knowledge

There are several gaps in our understanding of oHT that would benefit from future research. These are summarized in the Table and discussed below.

### Diagnosis

The diagnostic cut-off values for an exaggerated orthostatic pressor response and orthostatic hypertension are based on normative data rather than risk estimates. At least in some populations, more modest orthostatic increases in blood pressure are associated with higher cardiovascular risk. Additional data are required to refine diagnostic criteria in specific patient populations while avoiding overdiagnosis of orthostatic hypertension. The orthostatic measurement protocol also remains an important source of variability, including whether it starts from the supine or seated position, and the number and timing of readings while standing. As a result, reproducibility of the diagnosis remains an unresolved issue. It is reassuring that Hoshide et al. reported that identification of oHT based on home blood pressure readings was highly reproducible [33]. 

There is currently no consensus on who should be routinely screened, how often orthostatic blood pressure (BP) should be measured, or how serial orthostatic BP measurements should be incorporated into the management of orthostatic hypertension (OHT). Establishing diagnostic criteria using seated to standing blood pressure measurements will greatly facilitate the implementation of orthostatic measurement at home and in the clinic, and aid in the diagnosis and management of oHT.

### Pathophysiology

Sympathetic activation with standing exceeding requirements for blood pressure maintenance is a unifying feature of orthostatic hypertension. Yet, individual mechanisms mediating this response appear to differ qualitatively and quantitatively between populations. It is not yet known whether oHT is primarily driven by increased sympathetic outflow, heightened vascular responsiveness, arterial stiffness, impaired baroreflex buffering, or a combination of these mechanisms that differs across populations. Better mechanistic understanding could inform risk estimates and treatment decisions in orthostatic hypertension subgroups.

### Prognosis

The prognostic significance of oHT also remains incompletely defined. Most epidemiologic studies of orthostatic hypertension have been conducted in middle-aged and older populations with a high prevalence of hypertension. Consequently, whether adverse cardiovascular outcomes attributed to orthostatic hypertension are driven by the orthostatic pressor response itself or by the underlying burden of hypertension, arterial stiffness, and associated cardiovascular disease is uncertain. It is fortuitous that ongoing prospective clinical trials included orthostatic blood pressure measurements. Such efforts should continue. Ancillary trials could be conducted to determine which seated to standing threshold is equivalent to the current supine to standing consensus definition. This approach will take advantage of the valuable prognostic outcomes data already collected in the many trials that have measured only seated to standing blood pressures.

### Treatment

There is currently no evidence base for oHT-specific therapy. No randomized outcomes trials have tested whether targeted treatment of oHT improves cardiovascular, renal, or cognitive outcomes, and there are no validated recommendations for choosing among antihypertensive classes in patients with this phenotype. It also remains unclear whether antihypertensive treatments that fail to normalize elevated sympathetic activity [35] can contribute to oHT. Future trials should therefore test both efficacy and phenotype-specific safety to allow for an individualized, risk-based treatment approach in individuals with orthostatic hypertension.

Data on efficacy of targeting sympathetic activation with central sympatholytics or alpha blockers are limited. It seems unlikely that large randomized controlled trials using cardiovascular events as primary outcome will be forthcoming. Smaller mechanistic randomized placebo-controlled studies in cohorts defined by current consensus criteria would be helpful to determine if targeting sympathetic activity will selectively reduce oHT and have an impact on surrogate endpoints of end-organ damage (left ventricular hypertrophy, albumin/creatinine ratio).

## Summary & Conclusions

Orthostatic hypertension is increasingly recognized as a clinically meaningful blood pressure phenotype that reflects abnormal cardiovascular adaptation to standing. Although recent consensus definitions have improved diagnostic standardization, important uncertainties remain regarding optimal thresholds, measurement reproducibility, mechanistic heterogeneity, and the extent to which oHT independently predicts cardiovascular, renal, and cognitive outcomes. Current evidence suggests that excessive sympathetic vasoconstriction is the dominant mechanism in many patients, but arterial stiffness, impaired baroreflex buffering, and altered vascular responsiveness may contribute in different populations. Clinically, oHT may help identify individuals at higher risk who are not adequately characterized by seated blood pressure alone, but there is currently no evidence for oHT-specific treatment strategies or outcomes-based guidance. Further mechanistic, longitudinal, and interventional studies are needed to fill current gaps in our knowledge of oHT.

## Orthostatic Hypertension – Gaps in Knowledge and Actions Needed

### Diagnostic Criteria

- Refine orthostatic thresholds based on its prognostic impact on cardiovascular outcomes.
- Define diagnostic threshold on the specific patient populations (e.g., younger vs. older cohorts).
- Reach consensus about who should be screened for oHTN, and best implementation approaches.
- Establishing threshold criteria using seated to standing blood pressure measurements which would facilitate diagnosis and management.

### Pathophysiology

- Detailed mechanistic studies are needed in cohorts defined by current consensus criteria, to measure neural (sympathetic activity) and hemodynamic (venous pooling surrogates, cardiac output, vascular resistance).
- Comparison of these measurements in specific cohorts (e.g., young vs. old).

### Prognosis

- Continue to implement orthostatic blood pressure measurements in ongoing prospective trials.
- Ancillary studies to determine which seated to standing orthostatic blood pressure thresholds are equivalent to current diagnostic criteria.

### Management

- Mechanistic randomized controlled therapeutic studies targeting sympathetic activation to determine its effect on incidence of oHT and surrogates of end-organ damage.

## Key References

- Pasdar Z, De Paola L, Carter B, Pana TA, Potter JF, Myint PK. Orthostatic hypertension and major adverse events: a systematic review and meta-analysis. European Journal of Preventive Cardiology. 2023;30:1028–38. https://doi.org/10.1093/eurjpc/zwad158
  - ○ This is the first large systematic review and meta-analysis to demonstrate the clinical relevance of orthostatic hypertension as a hypertension phenotype. It pooled data across 13 trials, totaling over 55000 participants, to demonstrate a significant relationship between OHT and mortality, cardiovascular mortality, and stroke.
- Jordan J, Biaggioni I, Kotsis V, Nilsson P, Grassi G, Fedorowski A, et al. Consensus statement on the definition of orthostatic hypertension endorsed by the American Autonomic Society and the Japanese Society of Hypertension. Clin Auton Res. 2023;33:69–73. https://doi.org/10.1007/s10286-022-00897-8
  - ○ The consensus document is the first international attempt to harmonize diagnostic criteria of an exaggerated orthostatic pressor response and orthostatic hypertension, which up to then varied from study to study making it difficult to interpret the literature.
- Hirai T, Murai H, Sugimoto H, Mukai Y, Tokuhisa H, Ikeda T, et al. Differences in muscle sympathetic nerve activity between patients with orthostatic hypertension and those with orthostatic normotensive hypertension. Autonomic Neuroscience. 2026;263:103366. https://doi.org/10.1016/j.autneu.2025.103366
  - ○ This study provides direct evidence that individuals with OHT have higher resting MSNA than hypertensives with normal orthostatic blood pressure changes. This provides convincing mechanistic evidence that OHT is associated with a hyperactive sympathetic state and helps us gain a better understanding of the pathophysiology of OHT.
- Johansson M, Fedorowski A, Jordan J, Engström G, Nilsson PM, Hamrefors V. Orthostatic blood pressure adaptations, aortic stiffness, and central hemodynamics in the general population: insights from the Malmö Offspring Study (MOS). Clin Auton Res. 2023;33:29–40. https://doi.org/10.1007/s10286-022-00911-z
  - ○ The importance of this study is that it reinforces that a 10mmHg diastolic blood pressure rise on standing is a normal physiological response. It demonstrates that diastolic orthostatic changes are non-linearly related to aortic stiffness, supporting the consensus decision to define OHT using systolic criteria alone to improve clarity for future research in this field.

## Data Availability

No datasets were generated or analysed during the current study.
